# Hair Cortisol as a Biomarker of Chronic Stress in Community-Dwelling Adults With Longstanding Multiple Sclerosis

**DOI:** 10.1177/01939459251405618

**Published:** 2026-01-24

**Authors:** Nani Kim, Alexa K. Stuifbergen, Heather Becker, Theresa Swift-Scanlan, Nico Osier, Stephanie Buxhoeveden

**Affiliations:** 1 School of Nursing, The University of Texas at Austin, Austin, TX, USA; 2 School of Nursing, Virginia Commonwealth University, Richmond, VA, USA; 3 Accelerated Cure Project for Multiple Sclerosis, Waltham, VA, USA

**Keywords:** hair cortisol, chronic stress, chronic condition, multiple sclerosis

## Abstract

**Background:**

Chronic stress may play a significant role in disease progression in individuals with multiple sclerosis (MS), but traditional methods of measuring stress through salivary and blood cortisol have limitations. Hair cortisol has emerged as a promising objective measure for chronic stress assessment, but its application in those with MS remains understudied.

**Objective:**

To explore the feasibility of hair sample collection for cortisol analysis among community-dwelling individuals with longstanding MS (disease duration ≥20 years), including factors associated with participation and relationships between hair cortisol and self-reported measures, including perceived stress.

**Methods:**

In this cross-sectional study, 75 participants completed surveys and provided usable hair samples for cortisol analysis. Factors associated with participation and correlations among study variables were examined.

**Results:**

The completion rate for hair sample collection was 59.6% (n = 90). Of the samples returned, 83.3% (n = 75) were used in the analysis. Younger survey participants and those with shorter MS duration were more likely to provide usable hair samples. Hair cortisol showed no significant linear relationships with self-reported variables. In a multiple regression model to predict hair cortisol, employment was significantly associated with increased hair cortisol levels.

**Conclusion:**

Hair sample collection for cortisol analysis is feasible in community-dwelling individuals with longstanding MS. The lack of correlation between hair cortisol and perceived stress reflects the complexity of measuring chronic stress in MS, as in other populations. Future research may consider multi-method approaches to collect hair samples and explore relationships between hair cortisol, perceived stress, and health promotion variables.

## Introduction

Multiple sclerosis (MS) is a chronic, immune-mediated neurological disorder in which the body’s immune system mistakenly attacks the protective myelin sheath surrounding nerve fibers, leading to inflammation, neurodegeneration, and a wide range of symptoms caused by damage to the brain, spinal cord, and optic nerves.^1^ In 2004, a meta-analysis of 20 longitudinal studies demonstrated a strong association between stressful life events and subsequent symptom exacerbations,^2^ and in recent years, the impact of stress on individuals with MS has received attention due to its multifaceted effects on disease progression and health outcomes. A growing body of research is focusing on prolonged exposure to stress, because cumulative stress may exacerbate inflammation^3^ and increase the risk of gadolinium-enhancing lesions characteristic of acute relapses.^4^

### Measuring Chronic Stress in People With Longstanding Multiple Sclerosis

Chronic stressors in individuals with MS stem from a wide range of physical, emotional, and behavioral symptoms^5^ as well as social domains, which are associated with disease progression and the resulting disability that accumulates over time.^6^ In a qualitative meta-synthesis, Wilkinson and das Nair^7^ identified several MS-related factors that serve as sources of chronic stress. MS’s unpredictable trajectory, characterized by sudden relapses and stepwise progression, creates persistent difficulties in disease and symptom management. Disease progression worsens stress through accumulating functional limitations, such as gait disturbances and fatigue, impacting emotional distress and social isolation.^7^ Psychological comorbidities such as depression are highly prevalent in individuals with MS^8^ and may further intensify chronic stress.^9^ Economic pressures resulting from the direct and indirect costs of treatment can also contribute significantly to stress.^10^

The relationship between stress and MS may be particularly significant for individuals with longstanding MS (defined here as disease duration of more than 20 years since diagnosis), who face the cumulative effects of chronic stress. A postmortem study has revealed that hypothalamus-pituitary-adrenal (HPA) axis dysfunction may serve as a prognostic marker, with both hypoactivity and hyperactivity patterns observed.^11^ In that study, HPA axis reactivity, assessed with cerebrospinal fluid cortisol levels, was correlated with disease outcomes. Reduced HPA activity was associated with faster progression, including active inflammatory lesions and impaired remyelination, whereas hyperactivity in the HPA axis was correlated with slower progression. These opposing patterns may be understood through the relationship between high cortisol levels and enhanced anti-inflammatory and neuroprotective responses,^11^ suggesting the functional role of HPA reactivity in disease progression. The mechanisms underlying the chronic stress-MS relationship are not fully understood; however, because hypercortisolemia has been reported in MS cohorts in comparison with healthy individuals, glucocorticoid resistance and persistent low-grade inflammation are considered likely triggers for cortisol dysregulation.^12^ In older individuals, this dysfunction may be compounded by age-related factors, including immunosenescence, chronic inflammation, and declining remyelination capacity.^13^ It is thus important to assess cortisol levels and stress management as components of MS care.

Despite the growing body of evidence for the impact of stress in those with MS, there remains a gap in the objective measurement of chronic stress in this population. Although cortisol has been widely used as a biomarker of physiological stress levels in MS studies, most research has relied on saliva and blood samples,^14^ which have several limitations as an accurate reflection of cortisol levels over the long term. Both salivary and blood samples capture cortisol levels at a specific point in time and are susceptible to acute fluctuations driven by diurnal rhythms and situational factors, requiring repeated sampling to assess trends in stress exposure.^15^ In contrast, hair cortisol concentration (HCC) has emerged as a retrospective measure of long-term HPA axis activity.^16^ Each centimeter of hair represents approximately 1 month of cortisol exposure, and HCC requires only a single sample, substantially reducing participant burden in comparison with saliva or blood collections.^16^ A recent meta-analysis indicates that HCC levels are 22% higher in stress-exposed groups than in control groups.^17^ HCC demonstrates strong convergent validity, correlating with 24-hour urinary cortisol and longitudinal salivary cortisol profiles in diverse populations.^18,19^ The practical advantages of hair sampling, including noninvasive collection and room temperature stability, make HCC particularly suitable for chronic stress research,^15^ with established validity across older adults with chronic conditions.^20,21^ However, its application in MS populations remains limited, and current MS hair cortisol research is restricted to clinic-based collection.^22^ Given that people with MS live independently in communities, and considering the chronic nature of MS, self-administered hair collection at home represents a practical approach for future research using hair cortisol as a biomarker. Although underexplored in MS populations, the feasibility of self-administered hair sampling has been demonstrated in other populations, including community-dwelling adults, healthy college students, and dementia caregivers,^23,24^ supporting its potential applicability in MS research.

## Purpose

In this study, we investigated hair sample collection for cortisol analysis among community-dwelling individuals with longstanding MS. We examined key response metrics, including the completion rate of hair collection, the eligibility rate of hair samples, and the quality of obtained samples. We explored factors associated with participation in hair sample collection by comparing individuals who submitted samples with those who did not, and we examined the relationship between HCC as an objective measure for chronic stress and self-reported perceived stress, MS symptoms, health-promoting behaviors, and health outcomes.

## Methods

### Participants and Procedures

All study procedures were conducted in compliance with ethical guidelines and received approval from the Institutional Review Board at the University of Texas at Austin. The data for this cross-sectional analysis were derived from a single time point (2019; Year 23) of an ongoing 28-year longitudinal study examining individuals with MS. The initial cohort was recruited in 1996 from 2 Texas-based chapters of the National Multiple Sclerosis Society. At the time of initial recruitment, inclusion criteria required participants to have received a physician-confirmed MS diagnosis at least 1 year prior and to be living independently within the community. Consequently, all participants in this 2019 analysis had a disease duration of at least 24 years, representing a cohort with longstanding MS. A comprehensive description of the recruitment and methodological approach employed in the primary study has been published.^25^ Annual data collection was conducted via mailed surveys, which included self-report questionnaires with validated instruments. Participants remained in the study cohort unless they explicitly withdrew or were unable to continue due to death or transition to a nonindependent living arrangement.

In the 2019 data collection phase, we distributed a comprehensive survey battery, including self-report questionnaires and consent forms for hair sample collection, to 310 participants. Of these, 151 participants consented to hair sample collection and were mailed collection kits and detailed printed instructions. As an incentive, those who successfully submitted hair samples received a $25 gift card.

**Figure 1.**
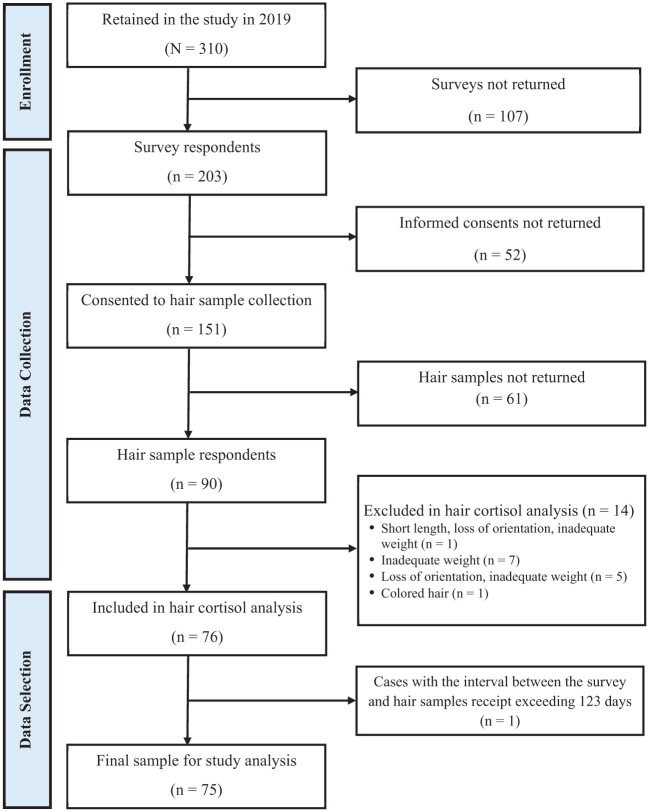
Participant flow diagram.

To evaluate the feasibility of hair sample collection, we examined 203 survey respondents, including 90 who submitted hair samples and 113 who did not (52 declined informed consent and 61 consented but did not return samples; see section “Data Analysis” for details). Following laboratory quality assessment, 14 samples were excluded for not meeting quality requirements, resulting in 76 valid samples. After excluding 1 participant whose hair sample was returned more than 123 days after survey completion (see section “Sample Selection for Study Analysis” for details), the final analytic sample was 75 participants. Figure 1 provides the participant and hair sample flow diagram, following the Strengthening the Reporting of Observational Studies in Epidemiology (STROBE) guidelines.^26^

### Hair Sample Collection

The hair collection kits, designed to ensure standardized sampling, included the following materials: hair ties, a sectioning grip clip (ie, crocodile clip), aluminum foil with stickers marking the scalp and distal ends, an antistatic bag, alcohol pads, and a printed copy of the protocol for sample collection and shipment, as described below. Our detailed protocol was based on methods previously used for at-home hair collection in other populations,^27,28^ with step-by-step written instructions and illustrative figures for all aspects of hair sample collection, including avoiding colored hair samples. A printed copy of the protocol was included in each participant’s hair collection kit, with instructions for (a) washing hair without conditioning products, (b) sterilizing cutting implements with alcohol pads or hot water, (c) applying a rubber band above the nape of the neck (lower posterior vertex region), (d) cutting hair to a minimum length of 1 cm and a minimum diameter of 0.25 inches, and (e) encasing the collected hair sample in the provided aluminum foil. Participants were given the option to either self-collect the sample or enlist the assistance of a professional hairdresser. Each participant returned one packet containing hair strands. Hair samples returned in 2019 were not processed prior to storage and were stored in individual plastic bags within an opaque index card box at room temperature until processing in November 2023. HCC has been shown to remain stable for several years under similar conditions in prior research.^16^

### Hair Sample Processing

Hair processing protocols for this study were adapted from Meyer et al^29^ and Ford et al.^30^ The hair was processed and pulverized in 3 stages before it could be resuspended as hair powder in assay diluent buffer prior to cortisol analysis using the Salivary Immunoassay Kit 1-3002 (Salimetrics LLC, State College, PA, USA).

#### Hair Sample Examination, Measurement, Washing, and Drying

During examination of the hair samples, we identified technical issues. First, 6 samples were returned with a loss of orientation. Despite instructions for participants to secure a rubber band proximal to the scalp and to place their hair samples with proper alignment of scalp and distal ends, the loss of orientation resulted from rubber band displacement or sample movement within the hair collection envelope and storage bag. Second, one sample was identified as colored hair. Lastly, hair samples from this study varied widely by weight and length, respectively ranging from 0.03 to 7.0 g and from <1 cm to 30 cm of hair proximal to the scalp in length. We therefore sought to standardize the input weight and length of hair prior to pulverization to 0.1 to 0.5 g and 4 cm of hair proximal to the scalp. Of the 90 hair samples provided, 76 met these criteria (with one 3 cm in length), and they were subsequently washed twice in 5 ml of 70% molecular grade Isopropanol at room temperature for 15 minutes. Hair samples were dried in a fume hood for 2 to 3 days to ensure complete evaporation of the isopropanol.

#### Pulverizing, Weighing, and Resuspension of Hair Powder

Each washed and dried hair sample was separately ground into a powder using a RETSCH Mill Jar (Catalogue No. 0841542; Thermo Fisher Scientific, Waltham, MA, USA) with grinding balls for 5 minutes at 25 Hz. The resulting hair powder was weighed, recorded, and used to normalize cortisol values as picograms cortisol per milligram of powdered hair, as in Ford et al.^30^ Each powdered hair sample was washed twice in 750 µl of molecular grade methanol for 24 hours under constant rotation. Both methanol washes were combined and evaporated in a Centrivap (Catalogue No. 10269710; Thermo Fisher Scientific) for 3 hours.

#### Resuspension of Pulverized Hair and Cortisol Assay

Powdered hair cortisol extracts were resuspended in 250 µl of salivary cortisol enzyme immunoassay diluent buffer and rotated at 28°C for 15 minutes. All samples were assayed in duplicate using the Salimetrics ELISA kit 1-3002 (Salimetrics LLC). The detection range of the kit is based on the concentrations of 6 standard curve samples run in duplicate that range from 0.012 to 3.0 µg/dl. *R*^2^ values for each curve were excellent (*R*^2^ = 1.00). Positive controls containing known high and low cortisol concentrations, and negative controls containing diluent buffer only, were included on each assay plate and run in duplicate. For colorimetric ELISA assays, conservatively acceptable intra-assay and inter-assay coefficients of variation (CVs) for replicate samples are considered at 20% to 25% or less. Impressively, 86.8% (66 of 76 participant hair samples) had CVs less than 10%, and 10 of 76 samples had CVs between 10% and 19%.

Cortisol plates were analyzed on the Spectromax i3x plate reader (Molecular Devices LLC, San Jose, CA, USA), and duplicate samples were averaged and calculated in micrograms per deciliter. Intra-assay and inter-assay CVs for 66 of 76 duplicate samples were less than 10%, and the remaining 10 duplicated samples had CVs that ranged between 10% and 20%. All cortisol values were converted to picograms cortisol per milligram of powdered hair as in Ford et al.^30^

### Sample Selection for Study Analysis

A subsequent step in sample selection for this study involved aligning the data collection timeline of the hair samples with previously completed self-report measures. Because survey packets and hair samples were collected via postal mail throughout the study, we implemented protocols to address potential temporal gaps between data collection points. First, we calculated the intervals between surveys and hair samples as follows. We used the survey receipt date as a time point in which participants completed their surveys and the returned date of their hair samples as the endpoint of hair cortisol measurement. Next, we set 4 months as a criterion for the intervals between 2 time points, following established evidence that a 4-cm hair segment is assumed to correspond to approximately 4 months of HPA axis activity.^31^ When the intervals exceeded 4 months (123 days), we assumed that the hair sample could be measuring cortisol levels outside of the timeframe of participants’ self-reported stress. Of the 76 hair samples initially assayed, the sample from one participant with an interval exceeding 123 days between these time points was excluded from study analysis. As a result, a final 75 cases were included for analysis.

### Measures

#### Background Information

Demographic characteristics, including age, biological sex, ethnicity, and education, were used from previous survey data. Current marital, employment, and clinical characteristics (eg, types of MS, current disease-modifying medications, current steroid treatment, and body mass index) were assessed via a survey for the present study. Perceived financial adequacy was examined with the Economic Adequacy Scale,^32^ with 8 items rated on a 4-point Likert scale that evaluate the extent to which financial resources are sufficient to meet monetary responsibility. Mean scores were calculated by averaging all answered items, ranging from 1 = *Not at all* to 4 = *More than adequately*, with higher scores indicating better financial status. Comorbid conditions were assessed with the Functional Comorbidity Index,^33^ which quantifies comorbidity as the sum of 18 medical diagnoses.

#### Hair Cortisol Concentration

HCC, derived from the proximal 4-cm segment of scalp hair, served as a biomarker of chronic stress over about 4 months.

#### Perceived Stress

The 10-item Perceived Stress Scale (PSS-10)^34^ was used to assess the impact of various situations on feelings and perceptions of stress over the preceding month. Participants rated each item on a 5-point Likert scale (0 = *never* to 4 = *very often*), with scores summed across all 10 items for a possible total from 0 to 40. Higher total scores indicate elevated levels of perceived stress. The PSS-10 has shown favorable reliability and convergent validity in community-dwelling older adults.^35^ In the present study, the PSS-10 exhibited good internal consistency, with a Cronbach’s alpha of 0.90.

#### Functional Limitations

Functional limitations attributable to MS were quantified with the 16-item Incapacity Status Scale (ISS).^36^ This self-report instrument assesses various domains of functional impairment, including ambulation and bowel/bladder function. Item responses are scored on a 5-point scale from 0 = *no limitations* to 4 = *maximum limitations*, with a possible total score of 64; higher total scores indicate greater perceived functional difficulty. Psychometric evaluations in MS populations have demonstrated adequate reliability and validity for the ISS.^37^ In the present study, the ISS exhibited good internal consistency, with a Cronbach’s alpha of 0.85.

#### Depressive Symptoms

The 10-item Center for Epidemiological Studies Depression Scale (CESD-10)^38^ was used to assess the frequency of depressive symptoms during the past week. Each item is scored from 0 = *rarely or none of the time* (less than 1 time per day) to 3 = *most or all the time* (from 5 to 7 days). Higher total scores represent greater frequency of reported depressive symptoms. Adequate psychometric properties of the CESD-10 have been supported across multiple studies focused on MS.^39^ Cronbach’s alpha for the CESD-10 in the present study was 0.81.

#### Health-promoting Behavior

Health-promoting behaviors were assessed with the Health Promoting Lifestyle Profile II (HPLP-II).^40^ This 52-item instrument measures the frequency of health-promoting activities across 6 domains: health responsibility, physical activity, nutrition, spiritual growth, interpersonal relations, and stress management. Each item is scored on a 4-point scale ranging from 1 = *never* to 4 = *routinely*. Higher scores indicate engagement in activities aimed at maintaining health and well-being. The HPLP-II has demonstrated favorable psychometric properties in studies involving older adults with MS.^41^ In the present study, the HPLP-II exhibited excellent internal consistency for the total score (Cronbach’s α = 0.92), with subscale reliability coefficients ranging from 0.72 to 0.87.

#### Perceived Social Support

We measured perceived social support using the Personal Resource Questionnaire (PRQ)-85 Part 2.^42^ The scale’s 25 items are scored on a 7-point scale from 1 = *strongly disagree* to 7 = *strongly agree*. Possible total scores can thus range from 25 to 175. Higher total scores indicate greater perceived social support. The PRQ-85 Part 2 has demonstrated sound reliability and validity in studies of older adults with chronic conditions.^43^ In the present study, Cronbach’s alpha was 0.93.

#### PROMIS Scales

The Patient-Reported Outcomes Measurement Information System (PROMIS) provides validated, person-centered measures to assess physical, social, and emotional health across populations with various health conditions.^44^ In the present study, we utilized PROMIS short forms to evaluate Pain Interference (6 items), Pain Intensity (3 items), Fatigue (7 items), and Sleep Disturbance (7 items). Higher scores on these scales indicate greater severity of the construct being measured. The psychometric properties of PROMIS scales have been well-documented across diverse populations,^44^ including people with MS.^45^ In our sample of 75, the PROMIS scales demonstrated excellent internal consistency, with Cronbach’s alpha ranging from 0.83 to 0.95.

#### Health Outcomes

The Medical Outcomes Study 36-item short form (MOS SF-36)^46^ was used to assess health-related quality of life. The MOS SF-36 includes 3 subscales that assess functional limitations (a) in physical activities due to health problems during the past week (role limitations due to physical problems), (b) in activities due to emotional problems (role limitations due to emotional problems), and (c) in social activities due to physical or emotional difficulties (social functioning). The raw scores of each subscale are summed, and the transformed scores are converted to a range with possible scores of 0 to 100 for comparability with a norm group; higher scores indicate greater functioning. The reliability and validity of the MOS SF-36 have been evaluated in studies of older adults with MS.^47^ Cronbach’s alpha for the 3 subscales in the present study ranged from 0.81 to 0.94.

Overall quality of life was assessed with a single-item measure. Participants were instructed to rate their current global quality of life on a 10-point scale ranging from 1 = *very poor* to 10 = *very good*. This approach to measuring quality of life, while brief, has been employed in previous research because of its simplicity and low respondent burden.^48^ And a single item was used to measure perceived health status on a 4-point Likert scale from 1 = *poor* to 4 = *excellent*.

### Data Analysis

A project manager and 2 research assistants checked the respondents’ returned surveys. If more than 15% of each subscale or total score was missing, we sent the respondents the pages with missing data and asked them to send us responses to those items. If missing items on a scale did not exceed 15%, we used mean substitution. Each research assistant independently performed data entry with IBM SPSS Statistics version 27 (IBM Corp, Armonk, NY, USA), followed by double-checking 20% of the responses.

Data analysis proceeded with 4 steps. First, descriptive statistics were computed to examine characteristics of study variables as hair collection metrics. We calculated the completion rate (the number of participants who successfully mailed their hair samples) and the eligibility rate (the proportion of hair samples used for analysis out of the total number of hair samples). Second, we conducted comparative analyses with independent *t* tests for continuous variables and with chi-squared tests for categorical variables, in order to identify differences in demographic and study variables (a) between participants who submitted hair samples (n = 90) and those who did not (n = 113) and (b) between participants whose hair samples were used for hair cortisol analysis (n = 76) and those whose hair samples were not, because of the hair samples’ poor quality (n = 14). For unequal variances in variables between 2 groups, we used Welch’s *t* test.

In addition, with our final sample of 75 cases, following the exclusion of the 1 case with a time interval of more than 4 months between survey completion and hair collection, we used Pearson’s *r* to identify relationships between HCC and the study variables. Multiple linear regression was conducted to predict HCC using perceived stress and covariates for the 75 participants. Subsequently, separate multiple linear regression analyses were conducted for progressive MS (primary, secondary, progressive-relapsing types) and nonprogressive MS (benign sensory, relapsing-remitting types). This distinction was made because individuals with progressive MS experience greater functional limitations than do those with nonprogressive MS.^49^ Covariates were selected on the basis of previous research and included age, BMI, cardiovascular diseases, and diabetes (ie, type 1 or 2).^17,50^ HCC was positively skewed (Fisher’s moment coefficient of skewness; *g*_1_ = 6.71); log-transformed HCC was 1.87. The significance level in all analyses was set at .05, two-tailed.

## Results

### Final Analytic Sample Characteristics

Table 1 shows the 75 participants’ demographic characteristics. Their mean age was 66.8 years, with a majority identifying as white (98.7%, n = 74) and female (93.3%, n = 70). Most presented with approximately 3 comorbid conditions and had been diagnosed with MS for an average of 31.4 years. Most had received at least 1 course of steroid treatment in the past, and only 2 reported currently undergoing steroid therapy. The HCC levels of these 2 participants were close to the mean HCC level (*z* = −0.20 and −0.12, respectively), so they were retained in the analyses. While the overall median of HCC levels before log transformation was 4.38 pg/mg (SD = 122.55; range = 0.81 to 970.64), the mean log-transformed HCC level was 0.80 pg/mg (median = 0.64; SD = 0.55; range = −0.09 to 2.99).

**Table 1.** Participants’ Demographic and Health Characteristics (n = 75).

Variables	n (%) or M ± SD
Age in years, M ± SD	66.81 ± 8.25
Biological sex, n (%)	
Male	5 (6.7)
Female	70 (93.3)
Ethnicity, n (%)	
White	74 (98.7)
Others	1 (1.3)
Latin heritage, n (%)	
Yes	2 (2.7)
No	73 (97.3)
Marital status, n (%)	
Married/living with significant other	54 (72.0)
Divorced/separated	7 (9.3)
Widowed	11 (14.7)
Never married	3 (4.0)
Employment, n (%)	
I work part time or full time for pay	11 (14.7)
I am unemployed due to disability	16 (21.3)
I am a full-time homemaker	10 (13.3)
I am retired	38 (50.7)
Years of school, M ± SD	15.00 ± 2.22
Education, n (%)	
No degree	3 (4.0)
Vocational training or certificate	4 (5.3)
High school (including GED)	21 (28.0)
Bachelor’s degree (including associates)	33 (44.0)
Graduate degree (master’s or doctoral)	14 (18.7)
Economic adequacy, item mean score, M ± SD	3.42 ± 0.67
Years with diagnosis, M ± SD	31.41 ± 5.53
Types of MS, n (%)	
Progressive MS	29 (38.7)
Nonprogressive MS	44 (58.7)
Unable to choose one answer/don’t know	2 (2.7)
Functional Comorbidity Index (counts)	2.89 ± 2.33
Body Mass Index,^a^ kg/m^2^	26.37 ± 5.28
Currently taking steroids, n (%)	
Yes	2 (2.7)
No	73 (97.3)
Steroid use over the course of multiple sclerosis, n (%)	
I have not taken steroids at any time	8 (10.7)
I have taken one course of steroid treatment	11 (14.7)
I have taken several (2-5) courses of steroid treatment	25 (33.3)
I have taken many (more than 5) courses of steroid treatment	31 (41.3)
Current disease-modifying therapies,^b^ n	
Copaxone or one of its generic equivalents	11
Ocrevus (Ocrelizumab)	9
Aubagio, Tecfidera, Lemtrada, or Tysabri	8
Betaseron, Avonex, Rebif, or Extravia	4
Perceived health status, M ± SD	2.71 ± 0.65
Poor (1), n (%)	2 (2.7)
Fair (2), n (%)	24 (32.0)
Good (3), n (%)	43 (57.3)
Excellent (4), n (%)	6 (8.0)
Quality of life (1 item), M ± SD	7.37 ± 1.75

Abbreviations: M, mean; MS, multiple sclerosis; SD, standard deviation; GED, general equivalency diploma.

a Two cases were excluded due to missing data.

b Multiple selections allowed.

### Hair Sample Collection

#### Hair Sample Collection Metrics

Both the survey and informed consent were returned by 151 participants for hair sample collection, 90 of whom returned hair samples, yielding a completion rate of 59.6% (90/151). Following our qualification process for hair cortisol analysis, 76 hair samples (84.4%; 76/90) were eligible for cortisol analysis, with the final 75 cases representing an eligibility of 83.3% (75/90).

#### Factors Related to Hair Sample Submission

There were no statistically significant differences in demographic characteristics or study variables between those who provided hair samples (n = 90) and those who did not (n = 113). A subsequent analysis comparing participants whose hair samples were initially included in the cortisol assay (n = 76) with those excluded (n = 14) yielded significant differences in years with MS diagnosis and age (see Table 2). Participants with valid hair samples reported a significantly shorter duration of MS diagnosis (*t*_88_ = 2.15, *P* = .049, mean difference = 5.2 years, 95% CI of mean difference: 0.04-10.43, *d* = 0.86). They were significantly younger (*t*_88_ = 2.47, *P* = .016, mean difference = 6.0 years, 95% CI of mean difference: 1.15-10.76, *d* = 0.72) in comparison with those with excluded samples.

**Table 2.** Comparison of Characteristics Between Participants With Included and Excluded Hair Samples for Cortisol Assay (n = 90).

Variables	Included in initial cortisol assay (n = 76)	Excluded from initial cortisol assay (n = 14)	χ^2^	*P*
	n (%)	n (%)		
Employment status				
Employed	11 (14.5)	2 (14.3)	.000	>.99
Unemployed	65 (85.5)	12 (85.7)		
Marital status				
Married (married/living with significant others)	53 (71.6)	8 (57.1)	1.16	.346
Not married (never married/divorced/widowed/separated)	21 (28.4)	6 (42.9)		
Types of MS				
Progressive (primary, second, progressive-relapsing)	29 (39.2)	5 (45.5)	.157	.748
Nonprogressive (benign sensory, relapsing-remitting)	45 (60.8)	6 (54.5)		
	M (SD)	M (SD)	*t*	*P*
Perceived Stress Scale	13.87 (7.35)	13.07 (8.27)	−0.37	.715
Age (years) in 2019	66.97 (8.32)	72.93 (8.26)	2.47	.016*
Economic Adequacy Scale	3.42 (0.66)	3.55 (0.50)	0.71	.478
Years of school completed	15.03 (2.24)	15.08 (2.69)	0.06	.951
Years with MS diagnosis	31.41 (5.49)	36.64 (8.81)	2.15	.049*
Incapacity Status Scale	17.42 (8.29)	18.64 (6.57)	0.52	.603
Functional Comorbidity Index (counts)	2.92 (2.35)	3.14 (1.46)	0.50	.622
CESD-10	8.14 (4.88)	9.02 (5.36)	−0.57	.569
BMI (kg/m^2^)	26.37 (5.28)	28.47 (7.26)	1.03	.317
HPLP-II Health responsibility	24.78 (4.71)	23.79 (7.03)	−0.51	.617
HPLP-II Physical activity	17.93 (6.18)	14.86 (4.19)	−1.78	.078
HPLP-II Nutrition	25.58 (4.82)	25.50 (6.19)	−0.05	.963
HPLP-II Spiritual growth	28.43 (4.96)	27.79 (7.04)	−0.41	.680
HPLP-II Interpersonal relations	28.67 (4.83)	30.00 (5.07)	0.94	.350
HPLP-II Stress management	24.24 (3.91)	24.36 (5.26)	0.10	.924
HPLP-II Total scores	149.76 (20.52)	146.29 (29.19)	−0.54	.589
PRQ-85 Part 2	141.98 (21.06)	147.25 (19.10)	0.87	.386
PROMIS Pain interference	14.57 (7.08)	11.79 (5.39)	−1.39	.167
PROMIS Pain intensity	6.97 (2.80)	6.14 (2.68)	−1.02	.312
PROMIS Fatigue	19.25 (5.84)	19.00 (5.35)	−0.15	.882
PROMIS Sleep	22.02 (6.95)	19.86 (7.60)	−1.05	.295
Perceived health status	2.72 (0.67)	2.79 (0.43)	0.45	.654
Quality of life	7.41 (1.76)	7.50 (1.74)	0.18	.857
MOS SF-36 Role limitations due to physical problems (Transformed score)	54.44 (31.65)	57.14 (22.18)	0.39	.701
MOS SF-36 Role limitations due to emotional problems (Transformed score)	74.78 (27.18)	74.40 (21.80)	−0.05	.961
MOS SF-36 Social functioning (Transformed score)	70.23 (28.50)	70.79 (20.13)	0.82	.413

Abbreviations: BMI, body mass index; CESD-10, 10-item Center for Epidemiological Studies Depression Scale; HPLP-II, Health Promoting Lifestyle Profile II; M, mean; MOS SF-36, Medical Outcomes Study 36-item short form; MS, multiple sclerosis; PROMIS, Patient-Reported Outcomes Measurement Information System; PRQ-85, Personal Resource Questionnaire-85; SD, standard deviation.

Following initial cortisol assay of 76 hair samples, one case was excluded from further analyses due to a time interval (>4 months) between survey completion and hair collection.

* *P* < .05.

### Relationships Between HCC and Study Variables

Table 3 presents the bivariate correlation matrix for all study variables. With the final 75 cases, log-transformed HCC was not significantly correlated with perceived stress (*r* = 0.15, *P* = .193). No significant correlations were observed between log-transformed HCC and demographic variables, health-promoting behaviors, PROMIS scales, or health outcomes at *P* < .05. Table 4 shows the results of a multiple regression analysis to predict log-transformed HCC with covariates and perceived stress. The overall model was not significant (*F*_7,64_ = 1.20, *P* = .32) and employment status was the only significant predictor in the regression model (*b* = 0.382, *t*_64_ = 2.08, *P* = .044), indicating that employed individuals showed higher HCC levels than did those who were not employed. Perceived stress was not a significant predictor of increased HCC (*t*_64_ = 1.87, *P* = .066). However, both employment status (β = 0.25) and perceived stress (β = 0.31) accounted for moderate variance in predicting log-transformed HCC, after controlling for other covariates.

**Table 3.** Bivariate Correlation Matrix (Pearson’s *r*, n = 75).

Variables		M	SD	Stress		Demographic						Illness-related				HPLP-II							Resources	PROMIS				Outcomes			
				1	2	3	4	5	6	7	8	9	10	11	12	13	14	15	16	17	18	19	20	21	22	23	24	25	26	27	28
Stress	1. Log-transformed HCC (pg/mg)	0.80	0.55	—																											
	2. Perceived Stress Scale	13.88	7.40	0.15	—																										
Demographic	3. Age in 2019	66.81	8.25	−0.07	−0.11	—																									
	4. Employment status (employed/unemployed)	—	—	0.22	0.04	−0.20	—																								
	5. Economic Adequacy Scale	3.42	0.67	−0.12	−0.27*	0.24*	−0.06	—																							
	6. Marital status (Married/not married)	—	—	−0.03	−0.19	0.06	−0.08	0.38***	—																						
	7. Years of school completed	14.99	2.23	0.03	−0.33**	0.18	−0.01	0.21	0.12	—																					
	8. BMI (kg/m^2^)^a^	26.48	5.22	−0.03	0.02	−0.21	0.08	−0.01	−0.06	−0.05	—																				
Illness-related	9. Years with MS diagnosis	31.49	5.58	−0.11	−0.20	0.36**	−0.06	0.21	0.13	−0.05	−0.22	—																			
	10. ISS	16.83	7.76	0.10	0.27*	−0.15	−0.06	−0.41***	−0.14	−0.04	−0.11	−0.04	—																		
	11. FCI	2.89	2.33	0.07	0.36**	−0.06	−0.06	−0.22	−0.22	−0.18	0.38**	−0.09	0.11	—																	
	12. CESD-10^b^	9.08	5.48	0.08	0.67***	−0.17	0.04	−0.28*	−0.04	−0.18	0.01	−0.20	0.30**	0.17	—																
HPLP-II	13. Health responsibility	24.94	4.65	0.06	0.08	0.23	−0.17	0.11	0.08	0.12	−0.03	−0.01	−0.10	0.29*	−0.11	—															
	14. Physical activity	18.07	6.19	0.08	0.23*	0.11	−0.02	0.21	0.01	0.09	−0.01	−0.04	−0.37**	0.18	0.07	0.42***	—														
	15. Nutrition	25.57	4.97	0.06	−0.16	0.19	−0.08	0.27*	0.28*	0.33**	−0.07	0.06	−0.21	−0.03	−0.23	0.49***	0.25*	—													
	16. Spiritual growth	28.39	4.96	0.22	−0.32**	0.13	−0.00	0.03	0.06	0.19	0.15	−0.03	−0.05	0.06	−0.51***	0.27*	0.12	0.41***	—												
	17. Interpersonal relations	28.61	4.90	−0.00	−0.24*	0.16	−0.16	0.19	0.29*	0.28*	0.05	0.11	−0.03	0.16	−0.34**	0.48***	0.28**	0.47***	0.71***	—											
	18. Stress management ^b^	24.30	3.98	0.13	−0.22	0.08	−0.25*	0.06	0.25*	0.14	−0.12	0.04	0.01	−0.07	−0.27*	0.42***	0.13	0.32**	0.51***	0.60***	—										
	19. Total scores ^b^	150.01	20.80	0.13	−0.13	0.21	−0.15	0.22	0.22	0.27*	0.04	0.02	−0.20	0.15	−0.32**	0.73***	0.58***	0.70***	0.71***	0.83***	0.66***	—									
Resources	20. PRQ-85 Part 2 total scores	141.89	21.13	0.02	−0.17	0.12	−0.12	0.30*	0.34**	0.13	0.03	0.08	0.05	0.06	−0.25*	0.34**	0.23*	0.41***	0.53***	0.79***	0.53***	0.67***	—								
PROMIS	21. PROMIS Fatigue	19.27	5.82	0.02	0.53***	0.05	−0.13	−0.47***	−0.18	−0.26*	0.09	−0.14	0.45***	0.35**	0.34**	0.10	−0.08	−0.14	−0.09	−0.09	−0.03	−0.09	−0.09	—							
	22. PROMIS Pain Intensity	14.61	6.98	0.11	0.41***	−0.04	−0.08	−0.38**	−0.19	−0.12	0.25*	−0.15	0.32**	0.55***	0.16	0.13	0.01	−0.23	−0.01	0.05	−0.05	−0.01	0.05	0.56***	—						
	23. PROMIS Pain Interference	6.88	2.73	−0.02	0.51***	0.03	−0.16	−0.41**	−0.21	−0.26*	0.15	−0.08	0.35**	0.48***	0.25*	0.10	−0.01	−0.21	−0.08	−0.06	−0.03	−0.07	−0.05	0.73***	0.85***	—					
	24. PROMIS Sleep Disturbance	21.89	6.63	0.08	0.28*	−0.04	−0.05	−0.09	−0.20	−0.00	0.19	−0.07	0.05	0.34**	0.40***	−0.14	0.16	−0.14	−0.26*	−0.23*	−0.33**	−0.19	−0.27*	0.16	0.29**	0.24*	—				
Outcomes	25. Perceived health status	2.72	0.66	0.09	−0.39***	0.13	0.01	0.35**	0.18	0.40**	−0.11	0.06	−0.46***	−0.33**	−0.41***	0.17	0.28*	0.45***	0.37***	0.29*	0.37**	0.46***	0.22	−0.36**	−0.40**	−0.43***	−0.24*	—			
	26. Quality of life	7.41	1.70	−0.06	−0.44***	0.09	−0.00	0.32*	0.08	0.27**	−0.04	0.09	−0.30**	−0.24*	−0.43***	0.23	0.25*	0.41***	0.44***	0.45***	0.39**	0.52***	0.42***	−0.44***	−0.30*	−0.42***	−0.35**	0.60***	—		
	27. MOS SF-36 Role Physical	54.31	31.22	−0.12	−0.50***	−0.04	0.11	0.41***	0.13	0.28**	−0.03	0.12	−0.54***	−0.24*	−0.29*	0.10	0.19	0.16	0.05	0.05	−0.01	0.14	−0.03	−0.71***	−0.52***	−0.67***	−0.17	0.37**	0.51***	—	
	28. MOS SF-36 Role Emotional	75.23	26.39	−0.09	−0.60***	0.14	0.02	0.38***	0.33**	0.18	−0.07	0.02	−0.49***	−0.39**	−0.31**	0.09	0.08	0.22	0.09	0.08	0.13	0.16	0.14	−0.55***	−0.45***	−0.54***	−0.20	0.42***	0.46***	0.60***	—
	29. MOS SF-36 Social Functioning	70.07	27.59	−0.09	−0.60***	0.10	−0.06	0.36**	0.21	0.35**	0.05	0.16	−0.41***	−0.27*	−0.35**	−0.04	0.15	0.27*	0.23*	0.20	0.14	0.23	0.20	−0.61***	−0.45***	−0.56***	−0.16	0.45***	0.57***	0.66***	0.68***

Abbreviations: BMI, body mass index; CESD-10, 10-item Center for Epidemiological Studies Depression Scale; FCI, Functional Comorbidity Index; HCC, hair cortisol concentration; HPLP-II, Health Promoting Lifestyle Profile II; ISS, Incapacity Status Scale; M, mean; MOS SF-36 Role Emotional, Medical Outcomes Study 36-item short form Role limitations due to emotional problems; MOS SF-36 Role Physical, Medical Outcomes Study 36-item short form Role limitations due to physical problems; MOS SF-36 Social Functioning, Medical Outcomes Study 36-item short-form role limitations in social activities due to physical or emotional difficulties; MS, multiple sclerosis; PROMIS, Patient-Reported Outcomes Measurement Information System; PRQ-85, Personal Resource Questionnaire-85; SD, standard deviation.

a Two cases excluded due to missing data.

b One case excluded due to missing data.

* *P* < .05. **P < .01. ***P < .001.

**Table 4.** Regression Analysis for Predicting Hair Cortisol (n = 72).

Model	*R* ^2^	*F*	*b*	95% CI for *b*	β	*t*	*P*
Outcome: Log-transformed Hair Cortisol	0.12	1.20					
1. Age in 2019			0.004	−0.013 to 0.021	0.06	0.44	.761
2. Employment status (1 = employed)			0.382	0.015 to 0.748	0.25	2.08	.044*
3. CESD-10			−0.019	−0.054 to 0.015	−0.19	−1.14	.258
4. BMI (kg/m^2^)			−0.001	−0.028 to 0.025	−0.01	−0.08	.938
5. Cardiovascular disease (1 = yes)			−0.071	−0.751 to 0.609	−0.03	−0.21	.835
6. Diabetes (1 = yes)			−0.202	−0.653 to 0.248	−0.11	−0.09	.374
7. Perceived stress			0.023	−0.002 to 0.048	0.31	1.87	.066

Abbreviations: BMI, body mass index; CESD-10, 10-item Center for Epidemiological Studies Depression Scale.

Three cases were excluded from a regression analysis due to missing data on BMI and CESD-10.

* *P* < .05.

Given the heterogeneity of MS disease courses, we conducted exploratory subgroup analyses by MS type (progressive and nonprogressive MS). As shown in Table S1, separate regression models were fitted for each subgroup; however, neither model reached statistical significance: progressive MS: *F*_7,18_ = 0.69, *P* = .68; nonprogressive MS: *F*_7,36_ = 0.64, *P* = .72; no predictor was significant in either subgroup.

## Discussion

In this study, we conducted the first assessment of hair sample self-collection for cortisol analysis in community-dwelling individuals with longstanding MS. We developed a standardized hair collection procedure and evaluated both the return rate of hair samples and their suitability for cortisol analysis. Given that our participants were older adults with longstanding MS, the likelihood of gathering hair samples from this population using flexible methods, including self-collection and assistance from hairdressers or others, is promising for hair cortisol analysis.

Of 151 participants who consented, the completion rate for hair sample collection in our study was 59.6% (90/151), which is lower than rates reported in previous studies of older African Americans (88.2%),^20^ people with dementia (76.9%),^21^ and parent caregivers (73%).^51^ It is possible that many constraints affected the lower completion rate, including hair issues (eg, insufficient or treated hair),^51^ mailing issues,^51^ procedural challenges,^20^ and different approaches depending on hair types (straight, curly).^16^ There are unique challenges in this population that may be attributable to common MS symptoms, such as fatigue and reduced hand dexterity,^52^ as well as hair loss related to disease-modifying therapy in females with MS.^53^

Despite the challenges, the quality of hair samples was acceptable in this study; 84.4% (n = 76) of the returned samples were suitable for cortisol analysis. This rate aligns with previous research, including the rates of 88.2% reported by Wright et al^20^ and 76.9% by Kim et al.^21^ Several factors may have contributed to our relatively high rate of usable hair samples. These include our detailed instructions for hair collection, assistance allowed during hair collection, and the trust between participants and the principal investigator developed over the course of a 23-year longitudinal study.

Nevertheless, self-collection methods present inherent limitations. Although we reached community-dwelling populations, we cannot verify adherence to proper instruction. Consistent with previous studies,^15,20^ we did find issues such as loss of orientation and insufficient hair quantity. To address these issues in people living with Alzheimer’s disease, Anderson et al^23^ have suggested dyadic self-collection procedures that allow caregiver assistance, incorporating additional collection procedures such as reminder calls, video instructions, and video conference meetings during collection, as well as increased compensation. Although we did not use such technology, adequate access to the internet and technological proficiency might also facilitate hair sample collection and increase the number of suitable hair samples for cortisol analysis. Addressing methodological variability in sample collection protocols may establish hair cortisol as a reliable and rigorous biomarker for evaluating stress-management intervention outcomes. Recent systematic reviews have examined the utility of HCC in understanding sleep quality and related disorders^54^ as well as psychological and neuropsychiatric interventions.^55,56^ Rogerson et al^56^ reported small-to-medium positive effects of stress-management interventions on cortisol levels; however, only 3 studies utilized HCC measurements. Across reviews, findings remain heterogeneous due to inconsistent assay procedures and varying methodological quality, highlighting standardization of sampling protocols, cortisol assays, and control of confounding variables as critical priorities for future research.

Our investigation into factors related to participation in hair sample collection revealed no significant differences in demographic data, MS symptoms, health-promoting behaviors, or health outcomes between participants who provided hair samples and those who did not. This may stem from the homogeneity of our study participants, who were predominantly white retired females participating in a 23-year longitudinal study. The finding that younger participants with shorter MS diagnosis periods were more likely to return usable hair samples for cortisol assay is unsurprising. Future research could investigate other possible differences between those who return usable samples and those who do not, with larger and more diverse groups of people with MS.

In our investigation of the relationships of HCC with study variables, employment status emerged as a significant predictor. Employed individuals demonstrated elevated HCC, suggesting that continued workforce participation in later life is associated with chronic stress and sustained HPA axis activation.^20^ Although a study in community-dwelling older adults found no relationship between employment status and HCC,^57^ our result indicates that occupational demands may contribute to chronic stress exposure in this population, consistent with findings in workers over age 50.^58^ The elevated HCC among employed participants may reflect the physiological burden of chronic stress from managing occupational responsibilities alongside MS symptoms and age-related challenges.

The relationship between perceived stress and HCC was not significant in this study, which is consistent with a meta-analysis of 72 studies.^17^ The relatively low perceived stress in our sample (median = 12 out of a total maximum score of 40) may have contributed to this lack of a significant relationship. However, further research on the relationship between perceived stress and HCC is needed. The stress-intensity hypothesis posits that an association between perceived stress and cortisol levels may occur only above a certain threshold for stress intensity, requiring constant or very frequent exposure to intense stressors.^17^ Evidence indicates a curvilinear relationship between HCC and perceived stress in diverse community samples.^59^ Another explanation may be a lag in HPA axis reactivity, meaning that although one may perceive oneself as stressed, the delay in HPA axis response and subsequent cortisol release may not be reflected in HCC.^60^ The discrepancy between perceived stress and HCC may also be attributable to differences in the interpretation of stress; the mind’s psychological response to a stressor may not align with the body’s chemical response.^57^ Furthermore, hair cortisol might be a better measure of global, nonspecific stress rather than stress in the specific domains that the PSS-10 measured.^61^ Future research should select a combination of self-report measures that target a broad range of stress responses.^62^

### Limitations

Several limitations of our study might influence the interpretation of our findings. First, we did not assess specific stressors, including their duration, severity, and controllability—crucial factors for comprehensive stress evaluation. The absence of such a detailed stressor assessment limited our ability to contextualize HCC and its relationship with perceived stress and variables related to health promotion. Second, we did not control for several factors known to influence cortisol levels, such as alcohol consumption, smoking habits, and detailed information about current medication use (eg, antidepressants) that might influence HCC.^50^ Other potential factors of hair care practice found in previous studies were also not investigated in the present study.^16^ The lack of control for these variables might have introduced confounding effects.

Third, the cross-sectional nature of our study precludes the examination of temporal relationships, so we could not infer causality in individuals with MS. In addition, the low representation of male participants (6.7%, n = 5) is a limitation. Although sex differences in hair cortisol have not been established in MS populations, studies of older adults (mean age >60 years) in Europe report higher hair cortisol levels in men than in women.^57,63,64^ The predominance of female participants in our study may have affected observed HCC levels, and our results should be interpreted with caution. Another limitation concerns the differing measurement windows between HCC and perceived stress. Hair cortisol analysis typically reflects a longer timeframe of stress exposure than does the PSS, which captures more recent experiences within the past month.^60^ However, synchronizing these measurement windows would have presented practical challenges, because collecting sufficient dried hair material (minimum 10 mg) for cortisol analysis would have been significantly more difficult with shorter hair segments. In order to better understand chronic stress, future studies should incorporate nuanced stress measures and longitudinal designs that account for factors influencing hair cortisol. Synchronizing measurement windows through self-reported chronic stress measures such as the Trier Inventory of Chronic Stress^65^ and longitudinal data collection using the PSS-10 could better improve chronic stress assessments in future research.

## Conclusions

Hair cortisol assessment using self-collected samples sent by mail was feasible in individuals with longstanding MS. Although the percentage of those who agreed to participate was lower than that has been reported in previous hair assay studies, the percentage of usable samples was similar to that in other studies. Employment status was associated with higher hair cortisol levels, but perceived stress had no statistically significant relationship with hair cortisol. These results support the need for future studies with a multi-method approach to measuring self-reported stress or with longitudinal methodology to better understand chronic stress in individuals living with MS for more than 20 years by considering the various factors that influence their stress experience.

## Supplemental Material

sj-pdf-1-wjn-10.1177_01939459251405618 – Supplemental material for Hair Cortisol as a Biomarker of Chronic Stress in Community-Dwelling Adults With Longstanding Multiple Sclerosis Supplemental material, sj-pdf-1-wjn-10.1177_01939459251405618 for Hair Cortisol as a Biomarker of Chronic Stress in Community-Dwelling Adults With Longstanding Multiple Sclerosis by Nani Kim, Alexa K. Stuifbergen, Heather Becker, Theresa Swift-Scanlan, Nico Osier and Stephanie Buxhoeveden in Western Journal of Nursing Research
