# Supplementary material for: Hair Cortisol as a Biomarker of Chronic Stress in Community-Dwelling Adults With Longstanding Multiple Sclerosis
Source: West J Nurs Res. 2026 Jan 24;48(4):357–70. doi: 10.1177/01939459251405618 (PMC12996369; doi:10.1177/01939459251405618)
Supplement: sj-pdf-1-wjn-10.1177_01939459251405618 – Supplemental material for Hair Cortisol as a Biomarker of Chronic Stress in Community-Dwelling Adults With Longstanding Multiple Sclerosis [file sj-pdf-1-wjn-10.1177_01939459251405618.pdf]

Supplementary Table S1. Subgroup Regression Analysis Predicting Hair Cortisol in Progressive and Non-progressive Multiple Sclerosis

Model	R^2	F	b	95% CI for b	β	t	p
Subgroup 1: Progressive type (n = 26)							
Outcome: Log-transformed Hair Cortisol	0.21	0.69					
1. age in 2019			0.008	-0.032–0.048	0.10	0.42	.68
2. Employment status (1 = employed)			0.412	-0.451–1.275	0.22	1.00	.33
3. CESD-10			-0.023	-0.097–0.050	-0.18	-0.66	.52
4. BMI (kg/m^2)			0.013	-0.029–0.054	0.14	0.63	.54
5. Cardiovascular disease (1 = yes)			-0.333	-1.765–1.099	-0.11	-0.49	.63
6. Diabetes (1 = yes)			-0.188	-1.192–0.816	-0.09	-0.39	.70
7. Perceived stress			0.035	-0.012–0.083	0.42	1.57	.13
Subgroup 2 : Non-progressive type (n = 44)							
Outcome: Log-transformed Hair Cortisol	0.11	0.64					
1. age in 2019			0.000	-0.024–0.023	< 0.01	-0.02	.99
2. Employment status (1 = employed)			0.357	-0.107–0.821	0.26	1.56	.13
3. CESD-10			-0.016	-0.060–0.029	-0.17	-0.71	.49
4. BMI (kg/m^2)			-0.019	-0.064–0.027	-0.15	-0.82	.42
5. Cardiovascular disease (1 = yes)			0.211	-0.713–1.135	0.08	0.46	.65
6. Diabetes (1 = yes)			-0.111	-0.714–0.491	-0.07	-0.38	.71
7. Perceived stress			0.014	-0.021–0.049	0.20	0.81	.43

Note. Three cases were excluded from a regression analysis for progressive type due to missing data on BMI or CESD-10; * $p < .05$; CESD-10 = 10-item Center for Epidemiological Studies Depression Scale; BMI = Body Mass Index.
